# Unruptured Myelomeningocele Closure Surgery: A Case Report

**DOI:** 10.7759/cureus.65940

**Published:** 2024-08-01

**Authors:** Flávia F Duarte, Natália M Dias

**Affiliations:** 1 Anesthesiology, Hospital Garcia de Orta, Almada, PRT

**Keywords:** pediatric neurosurgery, neonate, neural tube defect, myelomeningocele, pediatric-anesthesia

## Abstract

Myelomeningocele (MMC) is an in-utero closure defect of the posterior portion of the neural tube, and it is the most common neural tube defect (NTD) compatible with life. It is usually associated with other congenital malformations, such as hydrocephalus and Chiari type 2 syndrome. Therefore, the long-term outcome depends on early repair, and the surgery is urgently scheduled. Newborns with MMC are a special population that requires meticulous preoperative preparation to maintain hemodynamic stability during the procedure and a favorable outcome. In this case report, we describe the challenges of unruptured myelomeningocele closure surgery in a newborn with 12 hours of life. This special case emphasizes the importance of a multidisciplinary approach between anesthesiologists, neurosurgeons, and plastic surgeons to provide the best care to this subset of patients.

## Introduction

Neural tube defects (NTDs) are congenital spinal cord malformations that can be treated and occur at different degrees of severity. Worldwide, the prevalence of NTDs has declined in recent decades due to better maternal nutrition, folate supplementation, and prenatal diagnosis [1-2].

Myelomeningocele (MMC) is the most common NTD compatible with life and is often diagnosed prenatally. The incidence is around 0.44-1 per 1000 live births [2]. It results in an open neural placode covered by a thin membrane and cerebrospinal fluid located in the thoracolumbar or lumbosacral spine areas [3]. The most common presentation is a myelomeningocele of the lumbosacral spine. Patients with MMC present with a spectrum of impairments. More than 75% of infants have hydrocephalus, and many have Arnold-Chiari malformations that will require a ventriculoperitoneal shunt, usually performed after initial repair. Primary functional deficits are paralysis of the lower extremities, sensory loss, bladder, bowel, and cognitive dysfunction. The long-term outcome depends on early repair to prevent infection and spinal cord dysfunction [4-5].

Surgery is scheduled urgently a few hours after birth and consists of the closure of the neural tube, followed by the closure of the dura and the skin without tension. Preoperatively, it is critical not to allow rupture of the sac that covers the spinal defect, which will result in an increased risk of meningitis.

Neonatal anesthesia requires a proper understanding of neonatal anatomy, physiology, and hemodynamic specifications. Neonates are very susceptible to hypothermia, hypoglycemia, and desaturation during intubation attempts and have decreased compensatory mechanisms in response to blood loss. To practice safe general anesthesia, it is important to properly plan intubation and position, decide on the best anesthetic drugs and fluids based on weight, keep the newborn warm, and calculate the maximum allowable blood loss.

Therefore, this group of patients has both anesthetic and surgical concerns that require proper conduct and a multidisciplinary team. To the best of our knowledge, this is one of the biggest NTDs described in the literature. In this case report, we will present a review of the most important anesthetic and surgical considerations for the successful treatment of this rare case.

## Case presentation

**Figure 1:**
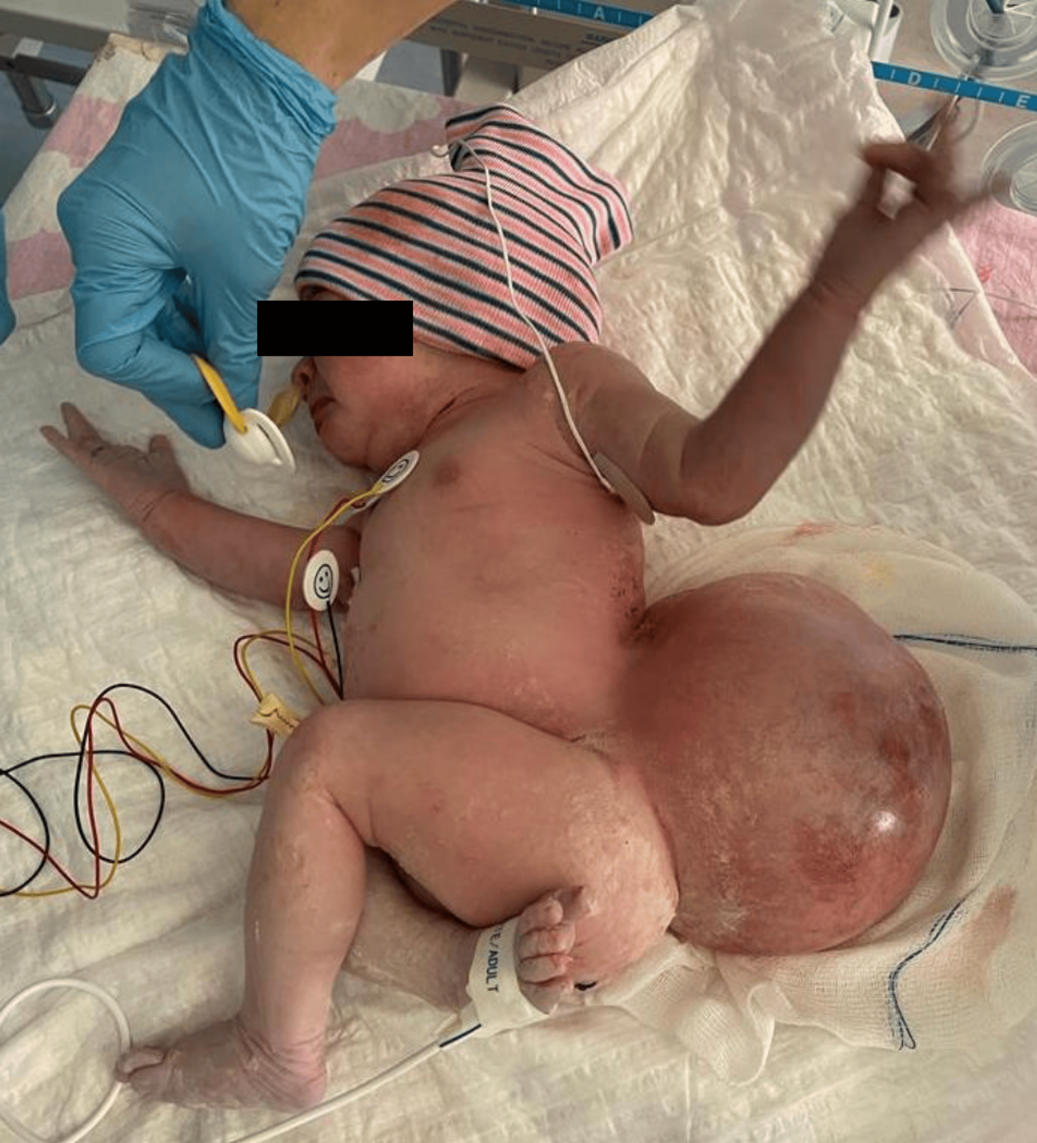
Lumbar myelomeningocele

A female newborn in supervised pregnancy with a prenatal diagnosis of myelomeningocele was born by elective cesarean section at 37 weeks and 3 days of gestation, weighing 4176 g. The APGAR score was 9/10. On neurological examination, the baby was active with spontaneous eye opening, pupils responded equally to light, and she had a flat anterior fontanel. She could move the upper limbs normally; however, there was hypotonia of the lower extremities, foot deformity, and only discrete movements of the legs. The intact myelomeningocele created a globular, fluctuating, and non-tender lumbar swelling with a diameter of approximately 15 cm. The skin surrounding the mass was red, with port wine stains, and was about to rupture at any time (Figure 1).

Transfontanellar ultrasound revealed corpus callosum hypoplasia and hydrocephalus, but no signs of Arnold-Chiari malformation. With the diagnosis of MMC with an impeding rupture, the baby was scheduled for urgent surgery to correct the neural tube defect.

On physical examination, she was hemodynamically stable, without the need for vasopressors, normopneic (respiratory rate of 45 cycles per minute), and had 96% saturation in breathing air. In pulmonary auscultation, the murmur was symmetric, with no adventitious breath sounds. Chest X-ray, electrocardiogram (ECG), and abdominal ultrasound were normal, as were preoperative hemoglobin concentration, coagulation tests, serum electrolytes, and glycemia. The echocardiogram did not show signs of cardiac or valvular dysfunction.

The neonate fasted for three hours for a breast meal and started a maintenance fluid (5% dextrose) at 10.5 ml/h in the intensive care unit (ICU). Twelve hours after birth, she was transferred to the operating room only for surgical repair of MMC. Neurosurgeons decided to evaluate the hydrocephalus after this surgery and the need for correction. 

In the operating room, heart rate, ECG, pulse oximetry, end-tidal carbon dioxide (EtCO_2_), minimum alveolar concentration (MAC) of anesthetic gases, neuromuscular depth by train-of-four monitor, rectal temperature, and urine output were monitored. We have access to invasive blood pressure and serial arterial blood gases through the umbilical arterial catheter. The baby had an umbilical venous catheter with two lumens and a 24 G peripheral catheter in her left leg. The operating room temperature was optimized at around 26 ºC, and active warming therapy was started prior to the induction of anesthesia. The baby was placed on a radiant warmer device and on a preheated convective blanket.

**Figure 2:**
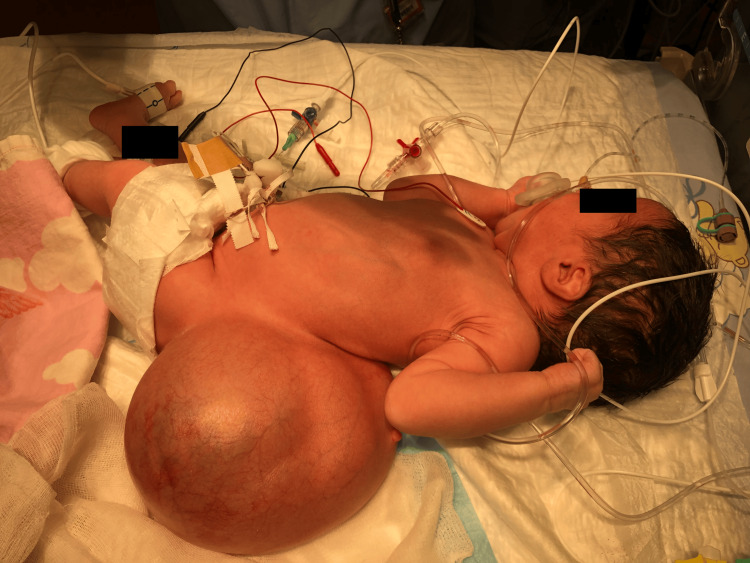
Newborn in supine position

Due to the large mass on her back, optimizing the supine position was difficult, making bag-mask ventilation and airway management challenging (Figure 2). 

Sevoflurane inhalation induction was the technique of choice. After confirmation that adequate preoxygenation with a face mask was possible, a bolus of 2 µg/kg of fentanyl and 0.6 mg/kg of rocuronium was administered before intubation. Due to the large size of the myelomeningocele, oral endotracheal intubation was performed in the lateral position. She was successfully intubated with direct laryngoscopy on the first attempt, and a 3.0 size uncuffed endotracheal tube was placed. The symmetric bilateral air entry was confirmed, and the tube was fixed. After intubation, 30 mg/kg of cefazoline was administered as a prophylactic antibiotic.

Right away, the newborn was placed in a prone position (symmetric air entry was reconfirmed), and rolls were applied under the chest and pelvis. The head was turned laterally and rested on a pillow.

Anesthesia was maintained with sevoflurane in a mixture of O_2_ and air, titrated according to the age-adjusted MAC value of 0.9-1. The baby was ventilated in pressure-regulated volume control mode with FiO_2_ between 28% and 35%, a tidal volume of 6 ml/kg, a respiratory rate of 48 cycles per minute, and a PEEP of 5. EtCO_2_ was kept between 35 and 40 and StO_2_ > 95% throughout the surgery.

During the intraoperative period, a fentanyl infusion was started at 1 µg/kg/h. Extra boluses of fentanyl (1 µg/kg) were given, for a total of three, justified by surgical stimulus. For fluid management, 5% dextrose was kept at 16 ml/h for maintenance therapy and warmed 0.9% sodium chloride was started at 5 ml/kg/h and gradually titrated (to a maximum of 10 mL/kg/h) according to surgical losses.

The surgery began with the gradual drainage of 750 ml of cerebrospinal fluid (CSF) from the lumbar collection with ultrasound support. Subsequently, the newborn’s systolic blood pressure fell below 50 mmHg with no response from three isotonic crystalloid boluses (10 ml/kg) and two 5% albumin boluses (20 ml/kg). So, noradrenaline infusion was started at 0.1 µg/kg/min and titrated to maintain systolic blood pressure around 60-65 mmHg (reaching a maximum of 0.25 µg/kg/min). At that time, arterial blood gas was collected without any relevant clinical findings. The result of arterial blood gas and the intraoperative monitoring trends are recorded in Table 1.

**Table 1:** Intraoperative monitoring

	Beginning of surgery	Immediately after drainage of CSF	After two hours of surgery	End of surgery
Arterial blood gas				
pH	7.35	7.37	7.42	7.38
pO_2_ (mmHg)	102	105	99	101
pCO_2_ (mmHg)	45	41	38	38
Haemoglobin (g/dL)	17.4	17.0	16.5	16.2
Glicemia (mg/dL)	89	104	112	115
Na^+^ (mmol/L)	132	132	137	138
K^+^ (mmol/L)	3.9	3.7	3.0	3.5
Intraoperative monitoring trends				
Systolic blood pressure (mmHg)	59	42	67	62
Heart rate (bpm)	152	189	164	157
Oxygen saturation(%)	96	97	97	98

**Figure 3:**
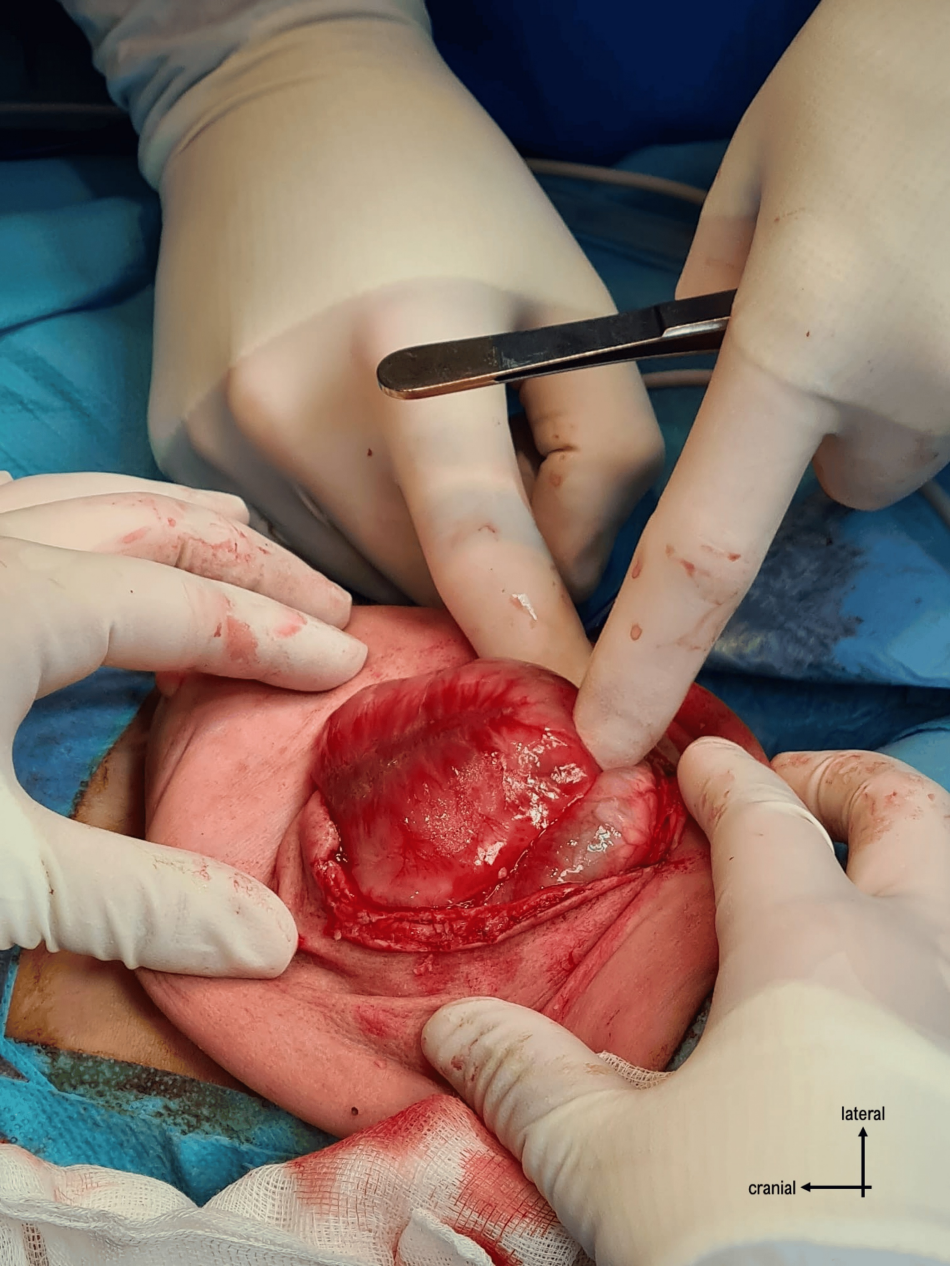
Identification of the placodium

**Figure 4:**
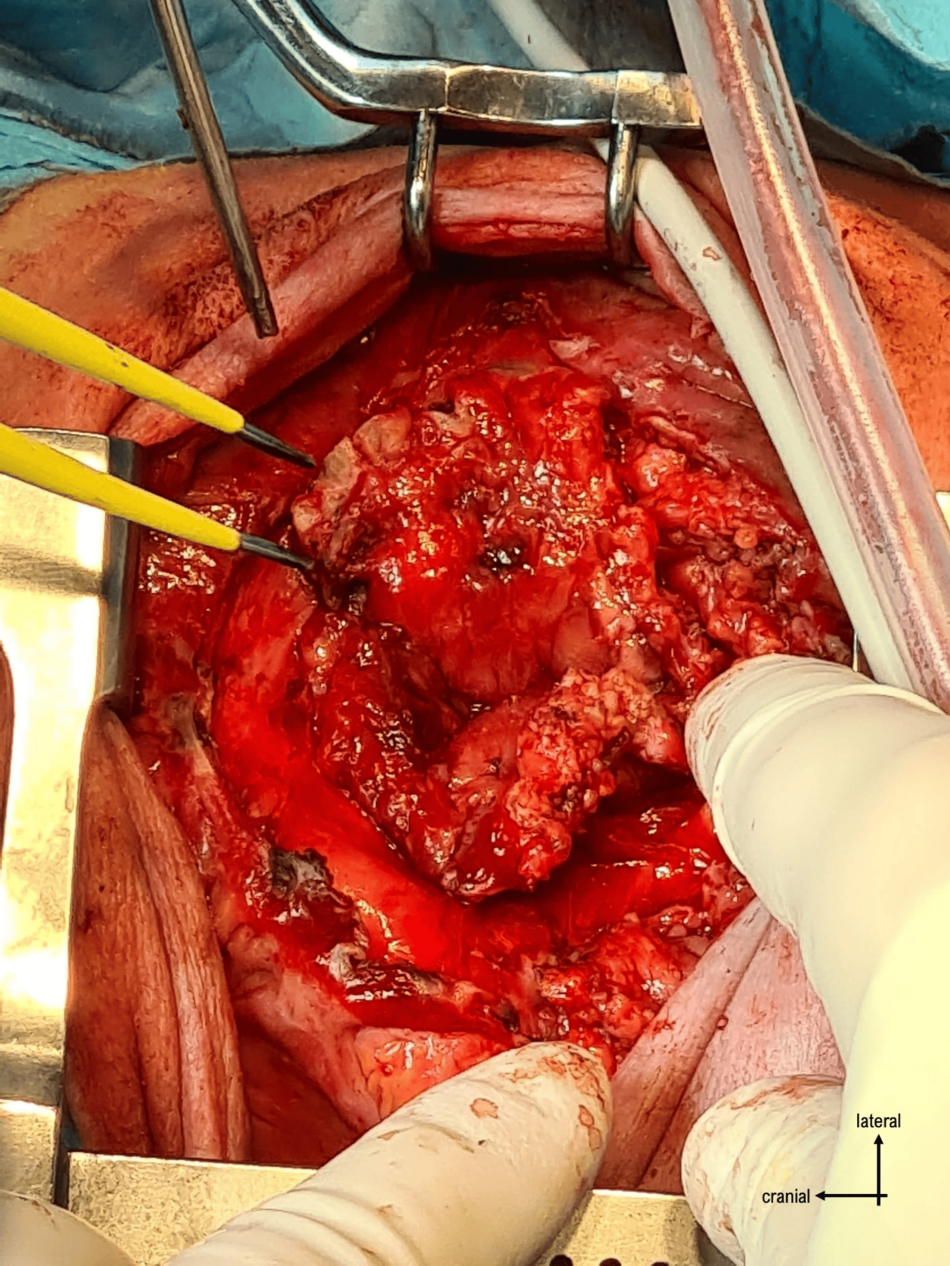
Dissection of the placodium

**Figure 5:**
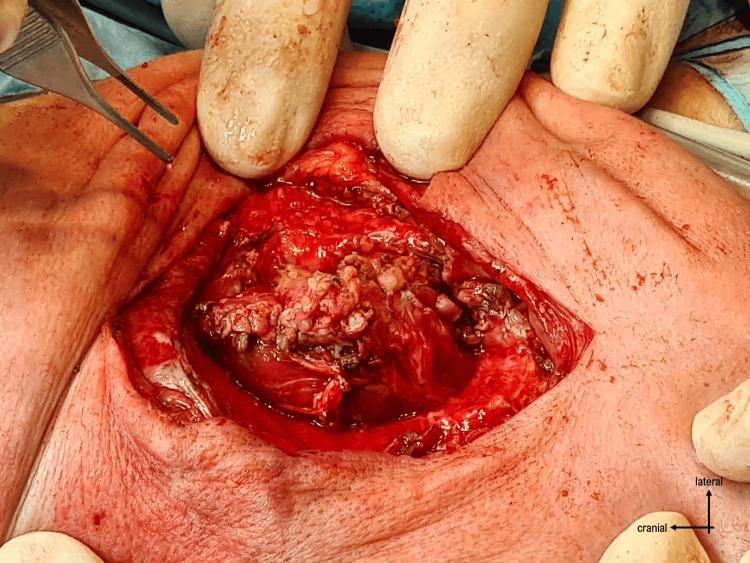
Closure of the placodium

The surgery proceeded with opening the skin through a vertical incision and identifying the plasmodium. It was dissected from the surrounding structures (Figures 3-4), and the neural tube was reconstructed by closing the plasmodium (Figure 5).

**Figure 6:**
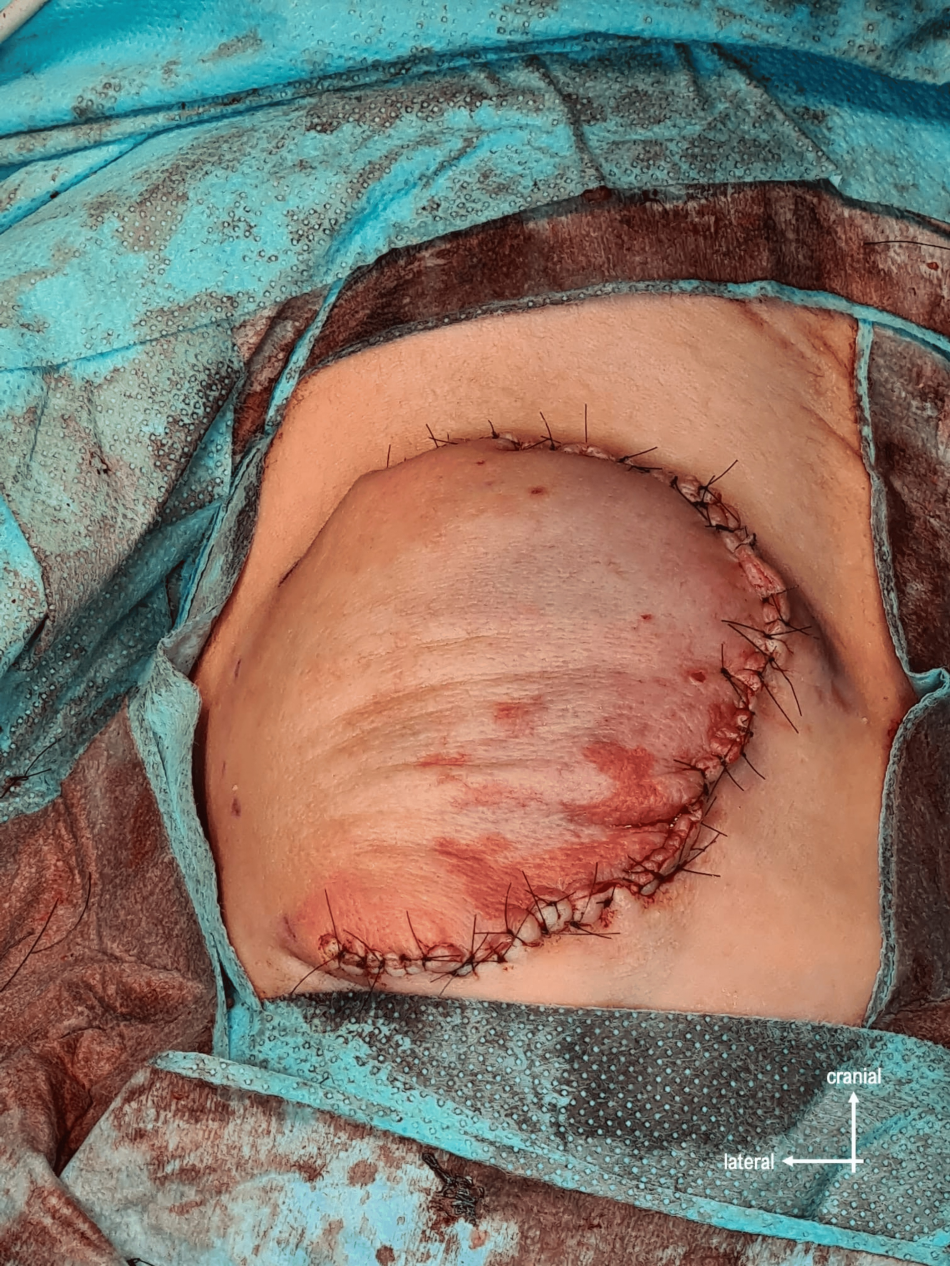
Closure of the skin

The defect was reinforced by two layers of dermis dissected from the excess skin, and then the skin was closed (Figure 6).

After two hours of surgery, a new arterial blood gas was collected that revealed a hypokalemia of 3.0 mmol/L (Table 1). A potassium correction of 0.2 mmol/kg/h was started and completed in two hours. Near the end of the surgery, the arterial blood gas was repeated, now showing no electrolyte abnormalities or any other changes (Table 1).

The baby was kept normothermic (with a core temperature variation between 36.5 °C and 37.7 °C) and normoglycemic throughout the surgery. The urine output was around 0.6 ml/kg/h, the estimated blood loss was approximately 40 ml (no blood transfusion was needed), and the duration of the surgical procedure was 240 min. There were no intraoperative complications. 

After the end of the surgery, the neonate was turned supine; it was confirmed by neuromuscular depth that there was no residual neuromuscular blockade; and the anesthetic gas was turned off. Due to hemodynamic stability, it was possible to stop the noradrenaline infusion. She was kept intubated and ventilated in pressure support mode, sedated with fentanyl infusion at 1.5 µg/kg/h, and transferred to the neonatal intensive care unit to be monitored in terms of hemodynamic and neurological status, ventilator difficulties, and brainstem herniation symptoms (such as high blood pressure with an irregular or slow pulse and loss of brainstem reflexes). She was extubated 24 hours after surgery without any complications.

## Discussion

Myelomeningocele, despite several neurological deficits distal to the defect and urologic and orthopedic symptoms, is the most common congenital anomaly that is compatible with life [6]. Surgery after a few hours of birth is important to restore normal spine alignment and reduce the risk of infection (the main cause of death during this period in this population) [7]. 

Central nervous system lesions could be associated with other organ abnormalities, such as congenital heart disease [8]. So, a preoperative evaluation of the cardiac, gastrointestinal, and genitourinary systems should be performed. Routine preoperative evaluation must include hematologic, serum electrolytes, biochemical, and coagulation profiles [9]. A complete neurological exam prior to surgery is mandatory to assess neurological deficits and the involvement of bulbar muscles.

Various difficulties are anticipated during surgery, such as positioning, airway and temperature management, and estimation of blood loss.

As the skin covering the NTD is very thin and threatens to rupture, adequate care is required during positioning. Patients are placed in the prone position, and the position and fixation of the tracheal tube, as well as the arterial and venous lines, must be ensured before and after turning the patient. A padded foam or jelly pad can be used to protect the ears, eyes, and application points. Practicing meticulous care during the prone position can prevent eye complications such as edema or corneal abrasions, unintentional extubation, or accidental dislodgement of access and monitoring lines [7].

In our case, due to the size of the myelomeningocele, tracheal intubation should be performed in a lateral decubitus position. Besides this, there were no other indicators of a difficult airway. However, the anticipated difficulty in airway management makes the availability of a well-established difficult airway algorithm and at least one type of video laryngoscope, flexible intubating scope, or rigid or semi-rigid scope necessary. The nasal or oral route could be performed according to the expert’s preference, as there is no evidence to recommend a specific one [10].

Intraoperative hemodynamic changes are another concern in the management of these cases. Low tolerance for third space and blood loss requires thorough control with adequate and timely replacement with warm fluids and blood [11-12]. Correction of large myelomeningoceles is more likely to involve high third space losses. Blood loss depends on the complexity and duration of the procedure. The timely administration of vasopressor perfusion according to needs helps to maintain adequate organ perfusion, minimizes spinal cord ischemia, and maintains hemodynamic stability during surgery. 

General anesthesia (GA) disturbs autonomic thermoregulation, and the redistribution of heat from the body core to the body periphery after induction of GA leads to an important drop in central body temperature. The risk of hypothermia is higher in newborns, and it can lead to apnea, bradycardia, hypotension, and acidosis [13]. Taking measures to preserve body temperature is particularly important in neonates with myelomeningocele because autonomic control below the defect level is abnormal. The measures to prevent hypothermia should start during transport. It should take place in an incubator, where the integrated temperature and radiant heat ensure temperature stability. Additionally, the operating room must be heated to a temperature around the thermoneutral range, and an effective active warning should begin before the induction of the surgery [14]. The irrigation solutions must also be heated to body temperature.

Follow-up care in an intensive care unit is required for close ventilatory, hemodynamic, and neurologic monitoring.

Although there are some case reports of interventions in newborns with defects in the neural tube without rupture, to the best of our knowledge, this is one of the biggest described in a newborn with 12 hours of life.

## Conclusions

Neonatal patients with MMC, due to the spectrum of associated deformities such as hydrocephalus, Arnold-Chiari malformation, congenital cardiac diseases, and genitourinary problems, are prone to both anesthetic and surgical challenges. On the other hand, the outcome of neonatal anesthesia depends on understanding the needs of this special population.

This case report highlights age-related pathophysiology and specific problems associated with neural tube abnormalities, the need for meticulous preoperative preparation, and continuous intraoperative and postoperative vigilance, adding important insights for the management of similar cases. The anticipated planning, with a multidisciplinary cooperative team of anesthesiologists, neurosurgeons, and plastic surgeons, allows for the avoidance of neurological and other complications that could occur during the preoperative period.
